# Perceptions and attitudes toward SLMTA amongst laboratory and hospital professionals in Ethiopia

**DOI:** 10.4102/ajlm.v3i2.233

**Published:** 2014-11-03

**Authors:** Adino D. Lulie, Tilahun M. Hiwotu, Achamyeleh Mulugeta, Adisu Kebede, Habtamu Asrat, Abnet Abebe, Dereje Yenealem, Ebise Abose, Wondwossen Kassa, Amha Kebede, Mary K. Linde, Gonfa Ayana

**Affiliations:** 1 Ethiopian Public Health Institute (EPHI), Ethiopia; 2 Shawnee State University, Portsmouth, Ohio, United States; 3 American Society for Clinical Pathology (ASCP), United States

## Abstract

**Background:**

Strengthening Laboratory Management Toward Accreditation (SLMTA) is a competency-based management training programme. Assessing health professionals’ views of SLMTA provides feedback to inform program planning, implementation and evaluation of SLMTA's training, communication and mentorship components.

**Objectives:**

To assess laboratory professionals’ and hospital chief executive officers’ (CEOs) perceptions and attitudes toward the SLMTA programme in Ethiopia.

**Methods:**

A cross-sectional descriptive survey was conducted in March 2013 using a structured questionnaire to collect qualitative data from 72 laboratory professionals and hospital CEOs from 17 health facilities, representing all regions and two city administrations in Ethiopia. Focus groups were conducted with laboratory professionals and hospital administration to gain insight into the strengths and challenges of the SLMTA programme so as to guide future planning and implementation.

**Results:**

Ethiopian laboratory professionals at all levels had a supportive attitude toward the SLMTA programme. They believed that SLMTA substantially improved laboratory services and acted as a catalyst for total healthcare reform and improvement. They also noted that the SLMTA programme achieved marked progress in laboratory supply chain, sample referral, instrument maintenance and data management systems. In contrast, nearly half of the participating hospital CEOs, especially those associated with low-scoring laboratories, were sceptical about the SLMTA programme, believing that the benefits of SLMTA were outweighed by the level of human resources and time commitment required. They also voiced concerns about the cost and sustainability of SLMTA.

**Conclusion:**

This study highlights the need for stronger engagement and advocacy with hospital administration and the importance of addressing concerns about the cost and sustainability of the SLMTA programme.

## Introduction

Laboratory services are an integral part of clinical decision-making and contribute to various aspects of health services, including the making of diagnostic and therapeutic decisions for patients, as well as disease monitoring and prevention.^1^ Historically, laboratories in developing countries have been under-resourced and marked by poor performance. These issues have fostered distrust in laboratory data amongst clinicians, reinforcing cycles of inadequate investment in laboratory systems. However, with recent emphasis on improving access to testing so as to meet the needs of expanded treatment and prevention programmes for HIV and other major diseases, the demand for diagnostics in resource-limited settings has increased substantially.^2,3^

Laboratory accreditation is used widely in developed countries to encourage and document improvements in the quality and reliability of test results. However, for laboratories in developing countries accreditation is a daunting challenge that only a handful of public laboratories in Africa have surmounted.^4^ As a result, the World Health Organization’s Regional Office for Africa has suggested that member countries improve the performance standards of their laboratories by implementing laboratory quality management systems (LQMS), establishing intermediate quality level goals and working toward accreditation in a stepwise manner.^5^

The laboratory network in Ethiopia is organised according to size and scope: health centre laboratories, hospital laboratories, regional reference laboratories and national reference laboratories. The Ethiopian Public Health Institute (EPHI), formerly known as the Ethiopian Health and Nutrition Research Institute, developed its first laboratory Master Plan in 2005 with a focus on building laboratory systems in the country. One of the strategic objectives was to expand and strengthen the national LQMS.

To accelerate the implementation of the LQMS, Ethiopia adopted the Strengthening Laboratory Management Toward Accreditation (SLMTA) programme, a competency-based management training programme that uses a series of short didactic courses and work-based learning projects to bring about immediate and measurable laboratory improvements and to prepare laboratories for accreditation.^6,7^ In 2010–2012, Ethiopia implemented the SLMTA programme in two cohorts with a total of 45 laboratories, comprising national and regional reference laboratories, referral hospital laboratories and district hospital laboratories throughout the country.^8^ Hiwotu et al. detail SLMTA implementation in Ethiopia, documenting substantial improvements to laboratory quality.^9^

Since its introduction in 2009, SLMTA has been implemented in 617 laboratories in 47 countries.^10^ However, no study has yet been published assessing the attitudes of healthcare professionals toward the programme. This study aimed to assess laboratory professionals’ and hospital chief executive officers’ (CEOs) perceptions and attitudes toward SLMTA programme implementation in Ethiopia in order to provide feedback for programme planning, implementation, communication and mentorship activities.

## Research methods and design

A cross-sectional descriptive study was conducted in March 2013 using structured surveys and focus group discussions. Seventeen facilities in total were selected purposively so as to ensure representation by geographic region and laboratory type, with one laboratory selected for each of the nine regions and two city administrations in the country, two national specialised hospitals in the capital city (Addis Ababa) and four additional hospitals in larger regions. All hospitals included in the survey had participated in the Ethiopian Hospital Management Initiative and hospital CEOs had received Master of Hospital Administration degrees through the Ministry of Public Health as part of this programme.^11^ A total of 72 people – 55 laboratory professionals (three to four per facility) and 17 hospital CEOs (one per facility) – were surveyed using questionnaires tailored to each group of respondents (Table 1). Hard and electronic copies of the questionnaires were distributed and all questionnaires were completed and returned to study investigators.

**TABLE 1:** Types of evaluation questions asked in surveys of laboratory professionals and hospital chief executive officers.

Laboratory managers and quality officers	Hospital chief executive officers
Outputs and impact of SLMTA	Awareness of the SLMTA programme
Challenges experienced during SLMTA implementation	Communication with laboratory staff during SLMTA implementation
Sustainability of the SLMTA programme	Resource allocation for SLMTA
Cost and availability of resources for SLMTA implementation	Sustainability of the SLMTA programme
Commitment of the Ministry of Public Health, facility management and laboratory staff for SLMTA implementation	-
Approach of SLMTA training	-
Importance of mentorship for SLMTA implementation	-

SLMTA, Strengthening Laboratory Management Toward Accreditation.

Two focus groups representing 12 of the 17 facilities were conducted, each with five laboratory quality officers and three laboratory managers (Table 2). In two-hour long interviews, investigators collected detailed data through open-ended questions and group discussions on the importance and sustainability of the SLMTA programme, the role of partners and the challenges facing the programme. Participants also shared their insights for future plans and implementation. Of the 16 focus group participants, three quality officers and one laboratory manager had also participated in the survey. The principal investigator, who had experience with qualitative data collection methods, facilitated the discussion, whilst the co-investigator, experienced in public health research methodology, captured the discussions through note-taking and audio recording.

**TABLE 2:** Profiles of laboratory professionals focus group participants.

Participant ID	Organisation	Sex	Age	Position
01	Regional Hospital Laboratory	Male	28	Quality officer
02	Regional Hospital Laboratory	Male	30	Laboratory manager
03	Regional Hospital Laboratory	Male	27	Laboratory manager
04	National Hospital Laboratory	Male	34	Quality officer
05	National Hospital Laboratory	Male	26	Quality officer
06	Regional Hospital Laboratory	Female	25	Quality officer
07	Regional Hospital Laboratory	Male	27	Quality officer
08	Regional Hospital Laboratory	Male	28	Laboratory manager
09	Regional Hospital Laboratory	Male	28	Laboratory manager
10	Regional Hospital Laboratory	Male	28	Quality officer
11	Regional Hospital Laboratory	Male	30	Quality officer
12	Regional Hospital Laboratory	Male	33	Laboratory manager
13	Regional Hospital Laboratory	Male	31	Laboratory manager
14	Regional Hospital Laboratory	Male	35	Quality officer
15	Regional Hospital Laboratory	Male	27	Quality officer
16	Regional Hospital Laboratory	Male	27	Quality officer

### Data analysis procedures

Quantitative data were entered and analysed using Microsoft^®^ Excel, after which descriptive statistics were used to present the findings. Qualitative data were analysed by transcribing and categorising responses.

## Results

### Survey

#### Regional-level laboratory managers’ perceptions of SLMTA

Data collected from the eight regional laboratory managers showed an overwhelming agreement on the importance of the SLMTA programme. All of the participants agreed that the programme had brought substantial improvements to the quality of laboratory services, one saying that SLMTA was ‘a catalyst for healthcare reform’ in Ethiopia. Respondents reported that stock outs of reagents were reduced, data management systems were improved and interruption of service resulting from equipment problems was minimised.

Respondents cited the commitment and participation of trained laboratory personnel, the national implementing unit and other implementing partners as major driving forces in the successful implementation of the SLMTA programme. All participating laboratory managers agreed that SLMTA has helped to accelerate improvements in laboratory networking, sample referral systems, competency of laboratory professionals through need-based trainings, laboratory equipment maintenance and electronic or paper-based data management.

Despite the significant benefits of the SLMTA programme, two of the eight respondents indicated that laboratory quality improvements were not as large as they had expected. The main challenges they faced in implementing the programme included high turnover of trained staff, inconsistent and inadequately-trained mentors and a lack of awareness regarding the importance of SLMTA at some facilities.

The respondents recommended further training for laboratory staff and sensitisation workshops for medical directors and hospital CEOs. For sustainable SLMTA implementation throughout the country, respondents suggested national strategic planning and empowerment of regional laboratories to oversee the programme in their respective regions.

#### Facility-level laboratory quality officers’ and managers’ perceptions of SLMTA

Of the 47 facility laboratory managers and quality officers surveyed, 40 (85%) viewed the SLMTA programme as being a critical step in the laboratory quality improvement process. All participants reported that SLMTA impacted every laboratory process and believed that positive and sustainable changes had occurred at all levels of the laboratory. They reported more confidence about the procedures to follow as well as a better understanding of the tests they performed, and felt that they were given more responsibilities for laboratory quality improvement processes.

Thirty-five (74%) of the participants reported satisfaction with the SLMTA training methods and that they gained important knowledge and experience. The remaining 12 (26%) respondents had various complaints about the training methods. For example, one respondent (Participant 03) reported that ‘the programme was launched without availing computers and providing basic computer skill training’ as there were no computers available at the training facility.

Half of the participating quality officers and laboratory managers related that, even though SLMTA demanded more resources than were currently available, improvements and changes were vital for their laboratories. They reported that the ministry planned to provide the resources necessary for accreditation preparation. The other half indicated that SLMTA’s value for accreditation preparation was low in comparison to the resources and time required to implement. One said, ‘[*w*]e were spending so much of our time preparing different documents that had no potential impact on the quality of laboratory services’ (Participant 01).

Thirty-five (74%) respondents indicated that they faced challenges resulting from a lack of commitment on the part of laboratory staff and management. The remaining 12 (26%) respondents said that staff and management commitment was not a problem in their facilities and that SLMTA implementation was facilitated by post-training orientation, close communication with management, scheduling of regular staff meetings and motivation of the staff and management.

All participants agreed that SLMTA had improved communication between laboratory staff and management and had led to measurable quality improvements. They reported that the most dramatic improvements were seen in reduced turnaround times, decreased equipment down times, new and functional data management systems and minimised supply lead times. Additionally, laboratory logistics information systems had been implemented and storage conditions improved.

Twenty-eight (60%) quality officers and laboratory managers indicated that there had been no regular mentor visits in their laboratories, either from EPHI or from implementing partners. All participants cited a lack of consistency amongst mentors and limited time for mentorship as being critical barriers to SLMTA implementation.

#### Hospital chief executive officers’ perceptions of SLMTA

From the survey of 17 hospital CEOs, 10 (59%) understood the importance, requirements and desired outcomes of the SLMTA programme, whilst seven (41%) were uncertain. The notable difference between the two groups was that the former worked more closely with laboratory management.

All 17 hospital CEOs agreed that the programme was resource-demanding and focused more on documentation than on actual laboratory testing. Eight (47%) believed that SLMTA was of insufficient value in their facilities given the significant amount of precious human resources consumed. Seven of these eight CEOs were from facilities that had scored zero stars at the exit audit. On the other hand, six (35%) CEOs whose facilities had improved and had scored one to three stars at the exit audit noticed the laboratory improvements and felt that the programme was valuable. Nine of the 17 hospital CEOs (52.9%) reported that they were so impressed with the programme that they were using the laboratory as a model for transforming their entire hospital system.

### Focus groups

#### Commitment, integration, output and impact of SLMTA on healthcare systems

Focus group members raised several issues during their discussions regarding outputs and impacts of SLMTA. They noted that they were proud of the results, which measurably improved quality of patient care. One participant shared his opinion:

‘SLMTA brings an irreversible laboratory revolution in our country.’ (Participant 07)

He continued by saying that laboratory professionals are the key to quality health services, as acknowledged by the Ministry of Public Health. Other respondents noted that the SLMTA programme promotes the standardisation of laboratory services, which contributes to customer satisfaction and confidence. They reported that there were now stronger and clearer laboratory policies and referral systems and that communication between laboratories had improved. Additional reported benefits of SLMTA included continuous improvement of services, opportunities for laboratories to conduct self-audits and movement toward compliance with national and international regulatory requirements.

Participants noted that SLMTA has been a catalyst for total health care reform and improvement in Ethiopia. One focus group participant noted the new integrated pharmaceutical logistics information system, which ‘improved the supply chain system of all pharmaceutical drugs in the country’ (Participant 05), as well as hospital reform initiatives aimed at improving the quality of the healthcare system, both of which were inspired by the SLMTA programme.

In addition, focus group participants generally agreed that SLMTA sparked an institution-wide revolution in safety and infection prevention practices throughout their hospitals. All laboratory staff were trained in basic laboratory bio-safety and post-exposure prophylaxis principles in facilities that implemented SLMTA. As one participant said:

‘Vaccination and safe waste disposal are coming true in our facility, [*a reality that seemed*] beyond our ability and capacity before.’ (Participant 16)

#### Cost and sustainability

A majority of the focus group participants agreed that the programme required more resources and time than they had anticipated. One participant said:

‘Even though I have no doubt of the importance of SLMTA for my country, the programme requires more resources which will hinder implementation in resource-limited situations.’ (Participant 04)

On the other hand, some participants did not notice the cost of SLMTA, since the programme is mainly funded by support from the government and implementing partners.

Participants expressed great concern about sustainability. All agreed on its importance, but noted that neither EPHI nor the implementing partners who initiated SLMTA in Ethiopian laboratories had designed strategies for sustaining the programme. Health facility management and the regional health bureau were not made aware of the need and cost of implementing and sustaining SLMTA. Furthermore, the roles of facility management and of the regional health bureau have not been clearly defined; this complicates the issue, since participants believe that sustaining the SLMTA programme is the responsibility of the government.

Participants agreed that sufficient action had not been taken to decentralise responsibility from the government to the management at their facilities, primarily because of a lack of regular communication between management at the facilities and EPHI. They recommended that all stakeholders, including laboratory management, facility management, EPHI and implementing partners, maintain close communication in order to share the responsibility of sustaining the SLMTA programme.

#### The role of mentorship and partnership

Participants agreed that regular mentorship as part of the SLMTA programme was important, but noted that this component was absent in some of their facilities. Major concerns included deficiencies in the number of trained mentors, uncoordinated planning between EPHI and regional facilities and lack of awareness of the importance of mentorship for quality management systems. Participants pointed out that the time allocated for mentorship was insufficient and that there were inconsistent skill levels amongst mentors. One shared her experience:

‘Even though we have limited mentorship services, we have tremendous knowledge and experience from mentors that improves our facility performance.’ (Participant 06)

The majority of the discussants agreed that implementing partners have played an important role in SLMTA execution. They have worked in collaboration with EPHI on capacity-building activities, such as renovation of laboratories, donation of modern laboratory equipment and provision of mentorship and basic training to laboratory staff on safety and quality management. One regional hospital representative shared:

‘The role of partners in the SLMTA programme was an essential piece for SLMTA implementation in our facility.’ (Participant 15)

However, some participants argued that the role of partners should be to build capacity and focus on short-term solutions, rather than to provide long-term support for SLMTA. One participant expressed concerns that partners have provided ‘a lot of support, but there was no joint planning between facilities and partners’ (Participant 10). Other participants wanted to take on more of the responsibility, saying:

‘We want the help of partners based on our plans and requirements.’ (Participant 09)

#### Challenges of SLMTA implementation

Common challenges identified included lack of commitment from facility management and laboratory staff, turnover of trained staff, insufficient regular mentorship, inadequate laboratory infrastructure, lack of awareness of the importance of the SLMTA programme and scarce resources. Other impediments included frequent equipment malfunction due to power interruption and lack of coordination between implementing partners and EPHI.

## Discussion

The attitudes of laboratory personnel in this study were generally supportive of the SLMTA programme. They recognised that SLMTA fundamentally restructured laboratory operations, heightened the confidence of laboratory professionals, improved data management systems and reduced turnaround times, equipment down times and supply lead times. Study participants agreed that the programme resulted in substantial improvements to the quality of laboratory services, laid the foundation for the implementation of an integrated pharmaceutical logistics information system and served as a catalyst for total healthcare reform and improvement in Ethiopia. Laboratory staff concerns focused on the issues of retention of trained staff, lack of regular mentor visits and resource requirements with regard to SLMTA implementation.

Hospital CEOs were more sceptical of SLMTA and raised concerns regarding programme costs and the prolonged process associated with implementation. In addition, many hospital CEOs did not have a clear understanding of the benefits of the SLMTA programme and most of those in hospitals whose laboratories remained at the zero-star level at the exit audit did not believe that the value of the improvements merited the human resources and time consumed. The roles of hospital management and regional health bureaus should be afforded greater attention in the implementation of SLMTA. Overall, the greatest concern was the cost and sustainability of SLMTA.

Previous studies have found similar results. Alkhenizan and Shaw conducted a systematic review of the global literature on the attitude of healthcare professionals toward accreditation.^12^ Two of the 17 studies identified focused on the laboratory personnel’s perceptions towards accreditation. Both studies found that most of the respondents preferred to work in an accredited laboratory because accreditation increased their confidence in the procedures they followed. However, the majority had concerns about the cost of accreditation and whether it had an effect on the quality of laboratory results.

The interface between laboratories and clinicians is important, as clinicians play a critical role in the use of laboratory services and test results; however, physicians and nurses were not interviewed in this study. An evaluation of their attitudes toward laboratory services before and after SLMTA implementation and their perspective on SLMTA in general would provide additional relevant information.

### Conclusion and recommendations

Laboratory professionals had a positive attitude toward SLMTA implementation in Ethiopia, seeing it as a driving force for substantial improvements in sample referral linkage, laboratory commodity management and laboratory data management systems. Half of the hospital CEOs were positive about the programme, whilst the other half, mainly those with low-scoring laboratories, were sceptical, highlighting the need for stronger engagement with hospital administration in order to address concerns about cost and sustainability. Future studies focusing on the cost-benefit of the SLMTA programme and long-term sustainability of results within SLMTA laboratories, as well as of the programme as a whole, would be beneficial.
